# Diversity and resilience of the wood‐feeding higher termite *Mironasutitermes shangchengensis* gut microbiota in response to temporal and diet variations

**DOI:** 10.1002/ece3.2497

**Published:** 2016-10-20

**Authors:** Ying Wang, Lijuan Su, Shi Huang, Cunpei Bo, Sen Yang, Yan Li, Fengqin Wang, Hui Xie, Jian Xu, Andong Song

**Affiliations:** ^1^ College of Life SciencesHenan Agricultural UniversityZhengzhouHenanChina; ^2^ Single‐Cell CenterCAS Key Laboratory of Biofuels and Shandong Key Laboratory of Energy GeneticsQingdao Institute of Bioenergy and Bioprocess TechnologyChinese Academy of SciencesQingdaoShandongChina; ^3^ Key Laboratory of Enzyme Engineering of Agricultural MicrobiologyMinistry of AgricultureZhengzhouHenanChina

**Keywords:** barcoded pyrosequencing, core microbiome, *Mironasutitermes shangchengensis*, resilience, temporal disturbance

## Abstract

Termites are considered among the most efficient bioreactors, with high capacities for lignocellulose degradation and utilization. Recently, several studies have characterized the gut microbiota of diverse termites. However, the temporal dynamics of the gut microbiota within a given termite with dietary diversity are poorly understood. Here, we employed 16S rDNA barcoded pyrosequencing analysis to investigate temporal changes in bacterial diversity and richness of the gut microbiota of wood‐feeding higher termite *Mironasutitermes shangchengensis* under three lignocellulose content‐based diets that feature wood, corn stalks, and filter paper. Compositions of the predominant termite gut residents were largely constant among the gut microbiomes under different diets, but each diet caused specific changes in the bacterial composition over time. Notably, microbial communities exhibited an unexpectedly strong resilience during continuous feeding on both corn stalks and filter paper. Members of five bacterial phyla, that is, Spirochaetes, Firmicutes, Actinobacteria, Tenericutes, and Acidobacteria, were strongly associated with the resilience. These findings provide insights into the stability of the gut microbiota in higher termites and have important implications for the future design of robust bioreactors for lignocellulose degradation and utilization.

## 1 Introduction

Termites have a high capacity for lignocellulose degradation and are believed to possess natural and efficient microscale lignocellulose bioconversion systems (Liu et al., 2011). They are generally classified into two groups: lower and higher termites (Ohkuma, 2008). Lower termites rely on bacteria, archaea, and cellulolytic flagellate symbiotic protozoa in their guts to digest cellulose (Brune, 2014). In contrast, higher termites lack flagellates and prokaryotes (Miyata et al., 2007) and are characterized by a purely eukaryotic gut microbiota (Kohler, Dietrich, Scheffrahn, & Brune, 2012). These microbiota are the major contributor behind the ability of higher termites to digest and utilize recalcitrant lignocellulose‐rich diets (Kohler et al., 2012).

The effects of diet and phylogeny of termites on their gut microbiota have been probed by past studies. *Firstly*, the diet has significant effects on the gut microbiome of both lower and higher termites (Boucias et al., 2013; Huang, Bakker, Judd, Reardon, & Vivanco, 2013). For example, the gut bacterial community of the lower termite *Reticulitermes flavipe* can be differentiated on the basis of grassy and woody diets as well as among different grassy diets (Huang et al., 2013). In the higher termite *Nasutitermes takasagoensis*, variations in the termite gut microbial community structures depend on the dietary composition (Miyata et al., 2007). These studies showed that Spirochaetes and Bacteroidetes are the main members of the bacterial community in the guts of both lower and higher termites. *Secondly*, structure of the gut microbiota is correlated with the termite phylogeny (Sanders et al., 2014; Wong, Chaston, & Douglas, 2013), although several microbiota are shared among numerous termite species. Therefore, host factors also play important roles in the gut microbiome composition. Most previous studies have investigated the termite gut flora of lower termites, whereas few have addressed those of higher termites. Notably, although several studies have demonstrated that the gut microbiota of termites are sensitive to different substrates, the temporal dynamics of the microbiota in lower and higher termites during a given feeding process remain elusive.

In this study, we characterized the temporal changes in the gut bacterial communities of the wood‐feeding higher termite *Mironasutitermes shangchengensis* under different diet disturbances by pyrosequencing the 16S rDNA amplicons. Three diets with different lignocellulose contents, that is, wood, corn stalks, and filter paper, were fed continuously to the termites, respectively. Samples were collected from termites fed with the wood diet on day 0 as a control for the other treatments (i.e., corn stalks and filter paper) because wood is the natural diet of termites. Compositions of the predominant termite gut residents were largely constant among the gut microbiomes under different diets, but each diet caused specific changes in the bacterial composition over time. Notably, microbial communities exhibited an unexpectedly strong resilience during continuous feeding on both corn stalks and filter paper. Members of five bacterial phyla were strongly associated with the resilience. These findings provide insights into the stability of the gut microbiota in higher termites and have important implications for the future design of robust bioreactors for lignocellulose degradation and utilization.

## 2 Materials and Methods

### 2.1 Termite collection and feeding

*M. shangchengensis* were collected from Jing Gangtai Nature Reserve (31°41′N latitude and 115°28′E longitude) in Henan, China. In order to keep them healthy and to avoid high temperatures or exposure to direct sunlight during transit, the termites were placed in containers with nest materials and wood from their natural environment until they were brought to the laboratory.

We tested 27 samples to determine the impacts of different diets and feeding times on the termite gut community structures. Three different lignocellulose content diets were tested, as follows: (1) wood (W, which comprised 18.5% lignin and 66.2% cellulose–hemicellulose); (2) corn stalks (C, which comprised 11.8% lignin and 62.3% cellulose–hemicellulose); and (3) filter paper (F, which comprised 99.99% cellulose). In laboratory conditions, approximately 1,500 robust worker termites were carefully removed from their containers without soil and were divided equally among three boxes. All of the feeding materials mentioned above were cut into small pieces and subsequently added to each box. The wood group (which served as the blank control) was used directly in the next step. The corn stalks and filter paper groups were subjected to three feeding time points (4, 7, and 10 days). Each group had four replicates, with the exception of the filter paper group, which had only three time points at 7 days due to low survival rate in captivity (Table S1). It should be noted that unlike lower termites, higher termites are less likely to survive outside their native environment. Few studies have addressed the feeding processes of higher termites, and 10 days is the longest survival time of higher termites in man‐made conditions. During the overall treatment process, all of the boxes were moistened periodically with sterile water and maintained in dark conditions at approximately 26°C.

### 2.2 Dissection of termites and DNA extraction

After treatment with different diets and time periods, 50 healthy *M. shangchengensis* individuals were selected from each group. Their exterior surfaces were washed with 70% ethanol and sterilized with distilled water before dissection. Next, their complete intestinal tissues (including all of the foregut, midgut, and hindgut) were dissected on ice using sterile forceps and needles. The tissues from different treatment groups were placed into different sterile 2 ml tubes. Next, 500 μl of phosphate buffered saline (0.2 mol/L, pH 7.4) was added to the tubes, before homogenization, and centrifugation at 3000 × *g* for 5 min at 4°C. The supernatant was recovered and used for DNA extraction.

The total intestinal microbial DNA of termites was extracted using an E.Z.N.A® Tissue DNA Kit (OMEGA, USA), according to the manufacturer's instructions. The concentrations of the DNA samples were determined using a spectrophotometer (Qubit™ Assays, Life Technologies, USA) at 260 nm, according to the recommended methods. Finally, 27 DNA samples were obtained, which were stored at −20°C until use.

### 2.3 PCR amplification of the V1–V3 region of 16S rDNA and pyrosequencing

To survey the microbial communities, the bacterial 16S rDNA V1–V3 variable regions were PCR amplified using a GeneAmp® PCR Systems 9700 (Applied Biosystems, USA). The forward (TGGAGAGTTTGATCCTGGCTCAG) and reverse (TACCGCGGCTGCTGGCAC) primers used for PCR were designed to amplify the V1–V3 hypervariable region of bacterial 16S rDNA. The primers included a unique sequence tag to barcode each sample. PCR amplification was performed in a 25 μl reaction volume, which comprised 12.5 μl of Go Taq® Sart Colorless Master Mix (Promega, USA), 1 μl of each primer, 1 μl of DNA template (10 ng/μl), and 9.5 μl double‐distilled H_2_O. The PCR reactions comprised an initial denaturation step at 95°C for 5 min, followed by 25 cycles of denaturation at 94°C for 30 s, annealing at 56°C for 30 s, and extension at 72°C for 30 s, with a final primer extension step at 72°C for 5 min. Each sample used a specific barcoded primer as a distinguishing factor. The PCR amplicons were visualized on 1.2% (w/v) agarose gel in 0.5 × TBE buffer (pH 8.3) using gel electrophoresis to confirm the amplification of appropriately sized products. According to the instructions provided with Agencourt AMPure XP beads (Beckman Coulter, USA), we recovered the purified and amplified PCR products, which measured ca 500 bp. The purified DNA concentrations were analyzed quantitatively using a Quant‐iT™ PicoGreen® ds DNA Assay Kit (Invitrogen, USA). The V1–V3 amplicons were sequenced using the pair‐end method with a 454 Life Sciences Genome Sequencer FLX Titanium (GS‐Titanium; 454 Life Sciences, Branford, CT, USA), according to the manufacturer's instructions.

### 2.4 Bioinformatics analysis of sequencing data

The pyrosequencing reads obtained from the sequencer were mainly processed with MOTHUR (Schloss et al., 2009). Raw reads were discarded if they: (1) had incorrect or unmatched primer sequences; (2) were <150 bp; (3) had an average quality score <30; or (4) had any ambiguous bases or homopolymers of <8 nucleotides. After the preliminary filtering process, the resulting sequences were denoised using the “pre.cluster” command (http://www.mothur.org/wiki/Pre.cluster) in MOTHUR. Chimera sequences were detected and removed by UCHIME (Edgar, Haas, Clemente, Quince, & Knight, 2011). To obtain the taxonomic assignments, all of the trimmed reads were clustered into OTUs with ≥97% similarity and aligned to the Greengenes database (McDonald et al., 2012) using the “claasify.seq” command in MOTHUR, with an 80% confidence threshold. The sequences associated with taxonomy classifications were classified according to the following levels: kingdom, phylum, class, order, family, genus, and unclassified if their levels were not clearly defined. The relative abundances of bacterial taxa were determined based on the number of sequences belonging to each OTU, which were calculated using R (version 3.1.2). All of the samples were rarefied, and only OTUs present in >90% samples were considered for further analysis.

Alpha diversity, including the analysis of Good's coverage, diversity estimators (Shannon's and Invsimpson), richness estimators (ACE and Chao1), rarefaction curves, heatmaps, and Venn diagrams, was calculated based on OTUs with ≥97% identity using the summary single command in MOTHUR (http://www.mothur.org/). Significant differences in Shannon's diversity index and PC1 values between treatment groups were determined using Sigmaplot with Wilcoxon's rank‐sum test. The phylogenetic beta diversity measure was used to investigate potential clustering of the microbial communities among different treatment samples, including unweighted and weighted UniFrac distance metrics analysis (Hamady, Lozupone, & Knight, 2010), where we used the OTUs from each sample in the analysis and visualization with QIIME software (Caporaso et al., 2010). To determine significant correlations along PC1 based on the OTUs and taxonomy, all of the data were normalized initially before removing the low‐abundance OTUs or phyla (variables that comprised >90% zeroes or mean relative abundance <0.01%), and the data were transformed into logarithm values (log2) and tested (Spearman |ρ| > .5, FDR *q* < 0.2) for significance using R (http://www.r-project.org). Wilcoxon's rank‐sum test was performed in R, and *p* < .05 was considered statistically significant.

## 3 Results

### 3.1 Estimated diversity and richness of the gut microbiota based on 16S rDNA sequencing data analysis

To investigate the bacterial diversity and relative richness in the gut of *M. shangchengensis*, we generated 656,564 paired‐end raw sequences with an average read length of 411 bp by parallel barcoded 454 pyrosequencing targeting of the 16S rDNA V1–V3 variable regions. After strict quality control and chimera checking, we obtained 315,719 high‐quality reads from 27 healthy worker samples, including 39,431 reads from the wood group, 138,972 from the corn stalks group, and 137,316 from the filter paper group. The remaining reads were binned into 9,285 operational taxonomic units (OTUs) after sequence alignment in the Greengenes database at 3% dissimilarity. In total, 3,842 of those 9,285 OTUs were defined as credible OTUs, which were present in over 20% of all samples and were consequently used for further analysis. All of the credible OTUs were assigned to 18 taxonomic phyla and 39 genera. In our study, Good's coverage was nearly 90%, which represented the overall composition of the gut microbiota among samples. The detailed indices are summarized in Table 1.

**Table 1:** Phylotype coverage and estimations of the richness and diversity within different treatment groups at 3% dissimilarity based on the 16S rDNA pyrosequencing analysis

Group	Raw reads	Reads analyzed	Good's Coverage (%)^a^	OTUs^b^	ACE^c^	Chao 1	Shannon	InvSimpson
Wood_0	20,087	9,858	88.92	2,199	4915.49	3731.63	6.70	314.44
Corn Stalks_4	16,472	8,081	86.15	2,197	4740.74	3647.65	6.75	266.79
Corn Stalks_7	22,431	10,738	88.64	2,566	4848.14	4086.43	6.96	389.29
Corn Stalks_10	32,687	15,924	91.73	2,997	5404.37	4614.46	7.00	443.36
Filters_4	27,804	13,043	90.23	2,697	5196.01	4335.48	6.74	228.62
Filters_7	25,124	11,460	88.21	2,993	5160.58	4640.99	7.22	533.18
Filters_10	25,818	12,691	90.59	2,510	5246.82	4056.79	6.80	359.86

a Good's coverage was determined as the estimated number of singletons in the population (n) compared with the total number of sequences (N), which was calculated as [1–(n/N)] × 100.

b Operational taxonomic units (OTUs) were defined at the level of 3% dissimilarity.

c Richness estimators (ACE and Chao 1) and diversity indices (Shannon's and InvSimpson) were calculated at the 3% dissimilarity level using MOTHUR.

**Figure 1:**
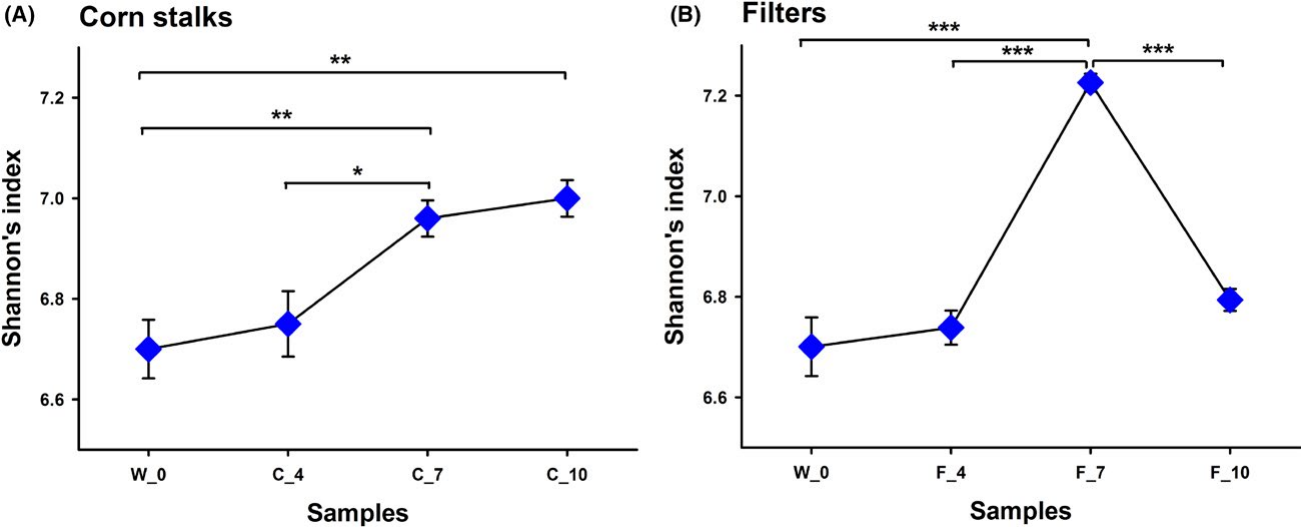
Shannon's index of Mironasutitermes shangchengensis gut microbiota ecosystems fed corn stalks (a) and filters (b) diets. Shannon's diversity index was used to estimate the diversity within different treatment samples, with calculations carried out at the 3% dissimilarity level. The data represent the mean ± SEM. *p < .05, **p < .01, ***p < .001, according to Wilcoxon's rank‐sum test.

We then used Shannon's diversity index to estimate the diversity of the total bacterial communities in termite guts. Shannon's diversity indices indicated that the bacterial diversity varied between individuals (Figure 1). In particular, under the corn stalks treatment, Shannon's diversity index of the W_0 group (original state) was lower compared with the C_7 group (feeding with corn stalks for 7 days) (*p* < .01, Wilcoxon's rank‐sum test) and C_10 group (feeding with corn stalks for 10 days) (*p* < .01, Wilcoxon's rank‐sum test), but there was no significant difference compared to the C_4 group (feeding with corn stalks for 4 days) (*p* = .59, Wilcoxon's rank‐sum test). These results demonstrate that the bacterial diversity increased with the corn stalks diet over time. Under the filter paper treatment, Shannon's diversity index of the F_7 group (feeding with filter paper for 7 days) was highest, compared with the W_0 group (*p* < .001, Wilcoxon's rank‐sum test), F_4 group (feeding with filter paper for 5 days) (*p* < .001, Wilcoxon's rank‐sum test), and F_10 group (feeding with filter paper for 10 days) (*p* < .001, Wilcoxon's rank‐sum test). This demonstrates that the diversity of the bacterial community in the F_7 group was more complex than that of the others and that it exhibits resilience when fed with filter paper.

Moreover, we used rarefaction analysis to estimate the richness of the gut microbiota. The steep slopes of the curves showed that a large proportion of the species diversity remained undiscovered in the samples. The rarefaction curves revealed that the slope was highest for the F_7 group, indicating that F_7 exhibited much higher bacterial richness than the other treatments. By contrast, the W_0 group had the lowest bacterial richness among the samples, thereby demonstrating that short‐term artificial feeding with corn stalks and filter paper increased the richness of the gut microbiota (Fig. S1). Despite significant individual variations among groups, those termites that were fed the wood, corn stalks, and filter paper diets still shared a large number of OTUs (3,382 OTUs), which showed that, although the different diets affected the structure of the gut communities, the core microbiome was relatively stable (Fig. S2). In addition, the Venn diagrams showed that, compared with the W_0 group, when fed both the corn stalks and filter paper substrates, bacteria from different feeding time groups shared a large number of OTUs (2,332 OTUs with corn stalks and 2,268 OTUs with filter paper) (Fig. S3A and Fig. S3B). These results indicate that the change in the feeding time affected the composition of the gut microbiota, but a stable microbiome core persisted across the complex microbial assemblages.

### 3.2 Structural composition of gut microbiota in the original state

**Figure 2:**
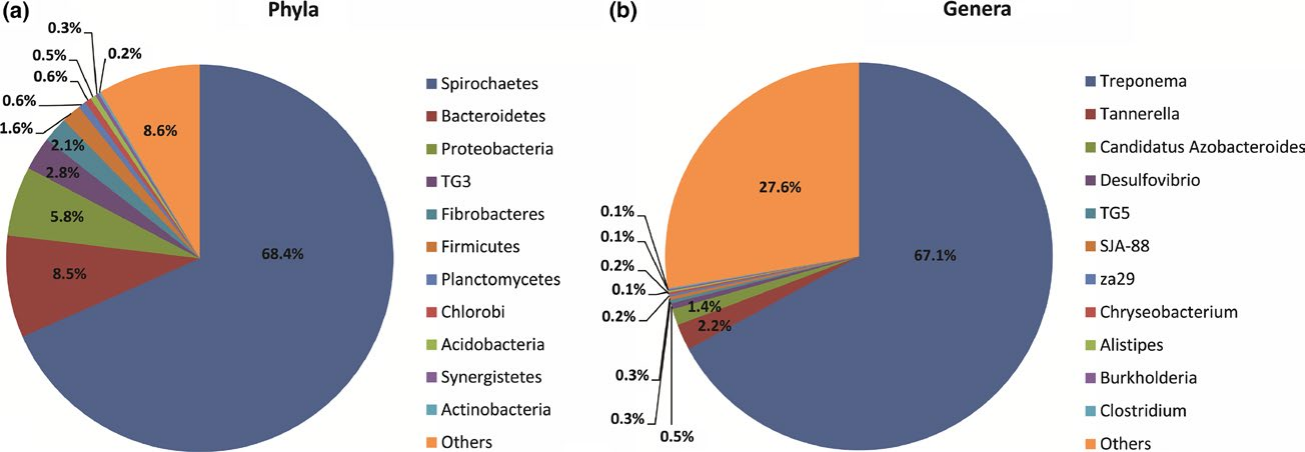
Phylogenetic abundance at the phylum (a) and genus (b) levels compared with individuals in the original state. “Others” represents relative abundance <0.1%

To investigate the variations in the gut microbiome composition after short‐term feeding with different diets, we assessed the bacterial community structures in the original state (the W_0 group). Because of the observed large deviations in the bacterial contents, bacteria that represented >0.1% of the relative abundance were designated as predominant bacteria, and those with >1% as the “core microbiota.” At the phylum level, 11 core bacteria phyla satisfied this definition. Among these core phyla, the predominant phyla were Spirochaetes (68.39%), Bacteroidetes (8.52%), Proteobacteria (5.83%), TG3 (2.77%), Fibrobacteres (2.10%), and Firmicutes (1.64%), followed by Planctomycetes, Chlorobi, Acidobacteria, Synergistetes, and Actinobacteria (Figure 2a). Most abundant phylum, Spirochaetes, was further classified into the classes Spirochaetes, Leptospirae, and Holophagae, and the families Spirochaetaceae, Leptospiraceae, and Holophagaceae. The core genera included *Treponema*,* Spirochaeta*, za29, and SJA‐88. Bacteroidetes, which was generally the second most abundant phylum, was represented by the classes Bacteroidia, Flavobacteria, and Sphingobacteria, and the families Bacteroidales and Rikenellaceae. At the genus level, 11 core bacterial genera were observed, among which *Treponema* (67.08%) was most prevalent. Two other predominant genera were *Tannerella* (2.18%) and *Candidatus* Azobacteroides (1.37%) (Figure 2b).

### 3.3 Structural comparison of gut microbiota among samples using multivariate statistics

**Figure 3:**
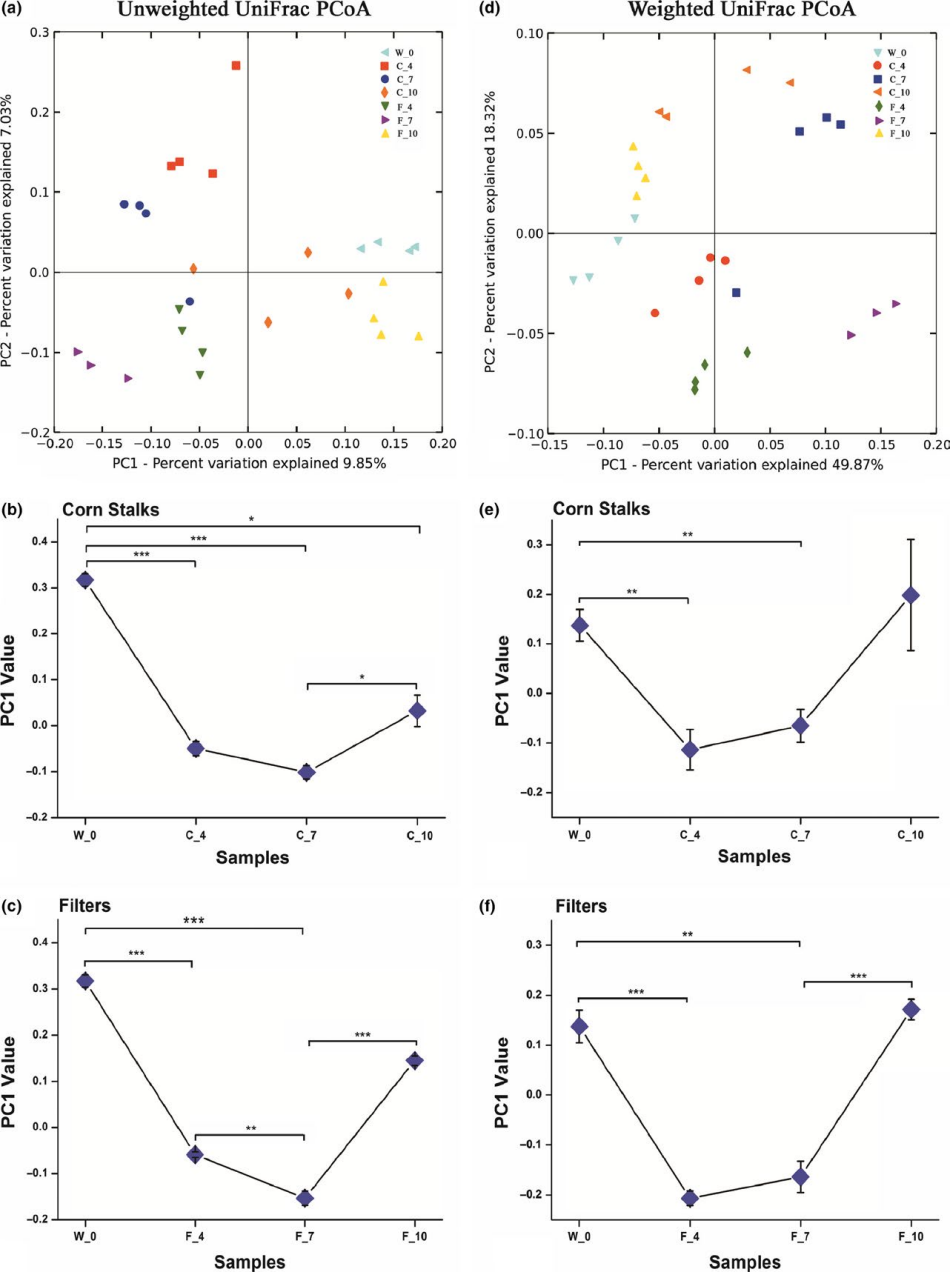
Unweighted and weighted UniFrac PCoA plots and the mean PC1 values of individual bacterial communities fed corn stalks and filter paper diets based on the unweighted and weighted matrices. (a, d) Principal coordinates analysis (PCoA) plots of the unweighted and weighted UniFrac distances, which were obtained using QIIME. (b, e) Comparison of the mean PC1 value with corn stalks as the substrate and different feeding times. (c, f) Comparison of the mean PC1 value with the filter paper as the substrate and different feeding times. The data represent the mean ± *SEM*. *p < .05, **p < .01, ***p < .001, according to Wilcoxon's rank‐sum test

To characterize the temporal changes in the microbiota structures after short‐term feeding with different substrates, we performed principal coordinates analysis (PCoA) based on both the unweighted and weighted UniFrac metric analysis. The microbiota from different treatment groups could be separated easily into seven distinct clusters based on the PCoA plot. The first principal coordinate (PC1) demonstrated the largest amount of variation and was strongly associated with microbiome change over time, which reflected the development of the microbiota structure throughout the temporal feeding process in the corn stalks and filter paper diets. The W_0 group was defined as the control group, which represented the original state of the termite gut microbiota, that is, without artificial feeding. Throughout the corn stalks feeding process, compared with the W_0 group, the C_4 group gradually moved away from the W_0 group (*p* < .01, *t*‐test) and was most distant on the 7th day (*p* < .01, *t*‐test), before moving back toward the W_0 group on the 10th day, at which point there was no significant difference (*p* = .62, Wilcoxon's rank‐sum test) (Figure 3a–c). Interestingly, this gut microbiota resilience was also found in the filter paper group, that is, after feeding for 4, 7, and 10 days, the W_0 group was significantly different from the F_4 (*p* < .001, Wilcoxon's rank‐sum test) and F_7 groups (*p* < .001, Wilcoxon's rank‐sum test). However, the original microbiota exhibited no significant difference from the F_10 group (*p* = .41, Wilcoxon's rank‐sum test) (Figure 3d–f).

### 3.4 Diet‐driven changes in bacterial abundance over time

**Figure 4:**
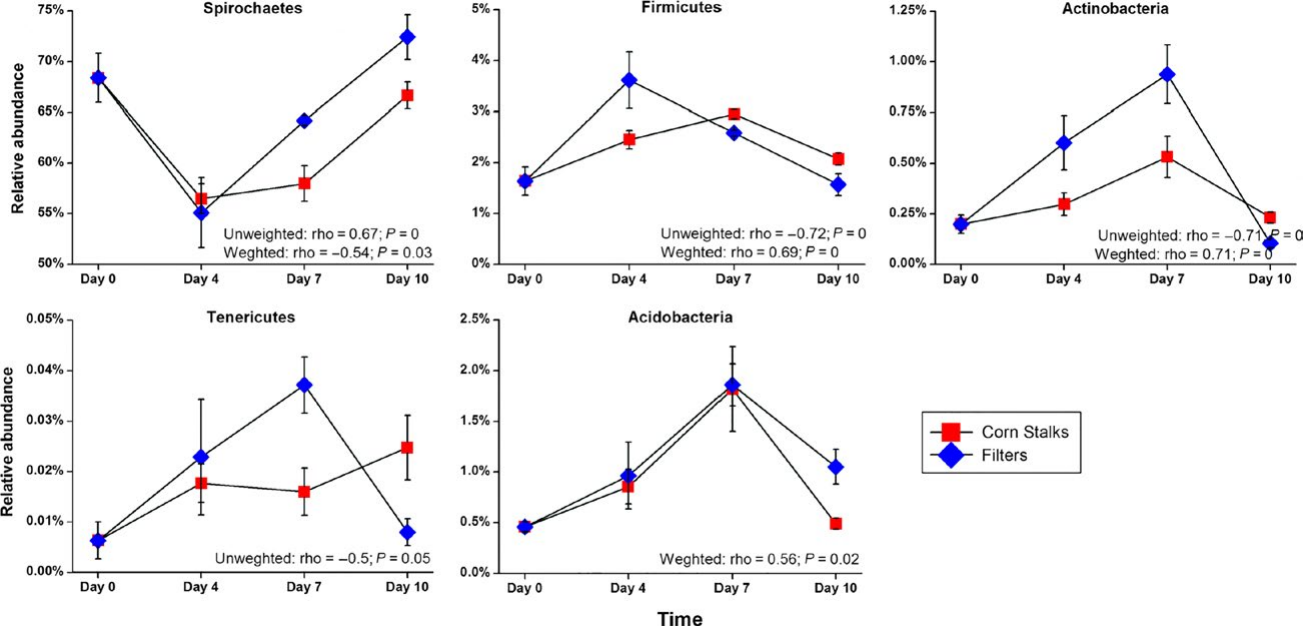
Relative abundances of the drivers of microbiota at the phylum level. All of these bacteria were significantly correlated with the PC1 coordinates based on the unweighted and weighted UniFrac PCoA (Spearman |ρ| > .5, FDR *q* < 0.2)

In order to identify OTUs that were closely related to the microbiota resilience observed, we tested the correlation between PC1 and all the OTUs in the samples from the corn stalks or filter paper groups (Figure 4). In this case, PC1 provides a quantitative descriptor of the temporal changes in the microbiota during the feeding process. At the phylum level, five phyla were identified as key components of the microbiota because changes in their abundance were significantly correlated with the PC1 coordinates, based on the unweighted and weighted UniFrac PCoA (Spearman |ρ| > .5, FDR *q* < 0.2) for both the corn stalks and filter paper diets. Tenericutes was only correlated with PC1 of the unweighted UniFrac PCoA, which emphasizes the acquisition/loss of unique taxa in this phylum over time. Acidobacteria was only correlated with PC1 of the weighted UniFrac PCoA, thereby suggesting that the relative abundance of most members of this phylum has changed throughout the dietary intervention. Furthermore, the major component of the variation between samples (PC1 in both the unweighted and weighted UniFrac PCoA) was related to changes in the abundance of Spirochaetes, Firmicutes, and Actinobacteria across samples. In reference to the original gut microbiota state (W_0 group), under either corn stalks or filter paper as substrates, Spirochaetes first decreased (after 4 days of feeding) before gradually increasing (after 7 days and 10 days of feeding) in terms of their relative abundance along PC1, whereas Firmicutes, Actinobacteria, and Acidobacteria increased initially (after 4 days and 7 days of feeding) before decreasing (after 10 days of feeding) along PC1. At the species‐equivalent level, 241 OTUs (at 97% similarity) were highly correlated with PC1 based on both unweighted and weighted UniFrac PCoA (Spearman |ρ| > .5, FDR *q* < 0.2), with nearly 49% of the OTUs assigned to the genus *Treponema* in the phylum Spirochaetes (Fig. S4 and S5).

Taken together, the results revealed that the termite gut bacterial community changed over time, and different diets significantly affected the compositions of the gut microbiota. In addition, the microbiota exhibited a strong resilience during continuous feeding on both corn stalks and filter paper, and bacteria with stable abundances during dietary disturbances in the corn stalks and filter paper diets might play important roles in the observed resilience.

## 4 Discussion

The termite gut microbiota ecosystem remains of interest, mainly due to their high capacity for degrading wood and other lignocellulosic substrates (Dietrich, Köhler, & Brune, 2014). Previous studies of the lower termite *Reticulitermes speratus* showed that Spirochaetes were the dominant member of the gut community, followed by Bacteroides, Firmicutes, Clostridia, and TG1 (Hongoh, Ohkuma, & Kudo, 2003; Ohkuma, 2008). Moreover, it was also shown that Spirochaetes and Fibrobacteres dominated the gut microbiota of the higher termite *Nasutitermes* spp. (Kohler et al., 2012). Many previous studies have focused on the gut bacterial community structure of lower termites, whereas the gut microbiota of higher termites is poorly understood. In the present study, we showed that the dominant bacterial phylum present in the gut microbiota of the wood‐feeding higher termite *M. shangchengensis* was Spirochaetes (nearly 63%), followed by Bacteroidetes, Fibrobacteres, and Firmicutes, as well as Proteobacteria, TG3, and Acidobacteria. The relative abundance of Spirochaetes was much higher than that in the lower termites mentioned above, which may be attributable to differences among species and differences within gut microbiomes (Dietrich et al., 2014).

The phyla present in the gut of lower and higher termites are largely similar, but there are distinct changes in the bacterial diversity when exposed to various substrates. Much evidence suggests that the bacterial diversity of the gut microbiota is essential for maintaining gut homeostasis (Boucias et al., 2013; Hu, Lukasik, Moreau, & Russell, 2014). In the gut of *Reticulitermes flavipes* termites, a diet of corn stover reduced the bacterial richness and diversity, compared with sorghum and two other woody diets (Huang et al., 2013). However, in the gut of *M. shangchengensis*, the bacterial richness and diversity both increased after feeding on corn stalks and filter paper, compared to wood. This may be related to differences in the host phylogeny: *M. shangchengensis* (higher termite) and *Reticulitermes flavipes* (lower termite) (Hongoh, 2010).

Monitoring the temporal dynamics allowed us to detect novel links between the gut microbiota and diet in termites. Several links between diet and the gut microbiota of termites have been established previously. For example, Miyata et al. showed that the gut microbial community of higher termites depends on the feed composition (Miyata et al., 2007). In addition, it was shown that there was no statistically significant difference after 7 days of feeding with lignin‐rich versus lignin‐poor cellulose diets (Boucias et al., 2013). However, to obtain a better understanding of the link between diet and the termite gut microbiota at spatial and temporal scales, we investigated the effects of different lignocellulose substrates (wood, corn stalks, and filter paper) and additionally considered the temporal dynamics of the gut microbiota community after different feeding times (0, 4, 7, and 10 days). Therefore, our results revealed the temporal effects of different diets on the gut bacteria community structure. Furthermore, by monitoring the temporal changes in both the alpha and beta diversity, we demonstrated that the higher termite gut microbiota have a high capacity to reverting to that of the termite in the wild state during continuous interventions with different diets.

Our most interesting finding was that the microbiota were resilient to perturbations when corn stalks or filter paper were used as substrates, which has rarely been reported in other termite gut microbiota studies. Furthermore, we identified the drivers that return the termite gut microbiota to the original state. Compared with the original state group (the W_0 group), when fed corn stalks or filter paper, the relative abundance of Spirochaetes decreased initially (after 4 days of feeding), before increasing gradually (after 7 days and 10 days of feeding), whereas Firmicutes, Actinobacteria, and Acidobacteria increased initially (after 4 days and 7 days of feeding), and then decreased (after 10 days of feeding). However, the abundance of Tenericutes differed between these two substrates throughout the short‐term feeding process. All of these bacterial phyla were significantly correlated with PC1, which indicated microbial resilience (Spearman correlation, |ρ| > .5, FDR *q* < 0.2). Based on these observations, we suggest that Spirochaetes, Firmicutes, Actinobacteria, Acidobacteria, and Tenericutes drive temporal changes in the termite gut microbiota.

It is well documented that the resilience response is particularly important when adapting to environmental change (Elmqvist et al., 2003), which may explain this resilience and the high stability of the higher termite gut microbiota community. Although artificial regulation did affect the termite gut flora for a short period, this change was not persistent in our work. During the dietary interventions, the intestinal flora eventually returned to its original stable state due to unknown regulatory mechanisms. Within the period of the dietary treatment, many members of the phyla Synergistetes, Elusimicrobia, TM7, ZB3, and Verrucomicrobia exhibited stable abundance, which may suggest that the core microbiota are linked to the resilience observed in the termite gut. These observations also suggest that future studies should validate the core microbiota by determining the resilience of specific microbiota to various perturbations.

In conclusion, our findings highlight the need for temporal scale explorations of the interactions between diet and termite gut microbiota. The resilience that we detected in this study provides novel insights into the artificial modification of the termite gut microbiome, the development of lignocellulose degradation bacteria, and their industrialized application in the future. The rational validation of those findings may have important implications for the future design of bioreactors used for biofuel production.

## Conflict of Interest

None declared.
