# Supplementary material for: Diversity and resilience of the wood‐feeding higher termite Mironasutitermes shangchengensis gut microbiota in response to temporal and diet variations
Source: Ecol Evol. 2016 Oct 20;6(22):8235–42. doi: 10.1002/ece3.2497 (PMC5108273; doi:10.1002/ece3.2497)
Supplement: Supplementary file 1 [file ECE3-6-8235-s001.doc]

| **Termite group** | ***M. shangchengensis*** | | | | | | |
| --- | --- | --- | --- | --- | --- | --- | --- |
| **Subjects** | Higher termites | | | | | | |
| **Substrate** | wood | corn stalks | | | filters | | |
| **Training time (day)** | 0 | 4 | 7 | 10 | 4 | 7 | 10 |
| **Subjects ID** | W_0 | C_4 | C_7 | C_10 | F_4 | F_7 | F_10 |
| **Sample parallel** | 4 | 4 | 4 | 4 | 4 | 3 | 4 |
| **Termite level** | workers | | | | | | |
| **Sampling site** | full gut | | | | | | |
| **Number of samples** | 50 | | | | | | |
| **Sampling position** | Jing Gangtai Nature Reserve  (31°41' N latitude and 115°28’ E longitude), Henan, China | | | | | | |

**Supporting Information**

**Table S1. Experimental design.** W, wood; C, corn stalks; F, filters; _0, original state; _4, feeding for four days; _7, feeding for seven days; _10, feeding for ten days


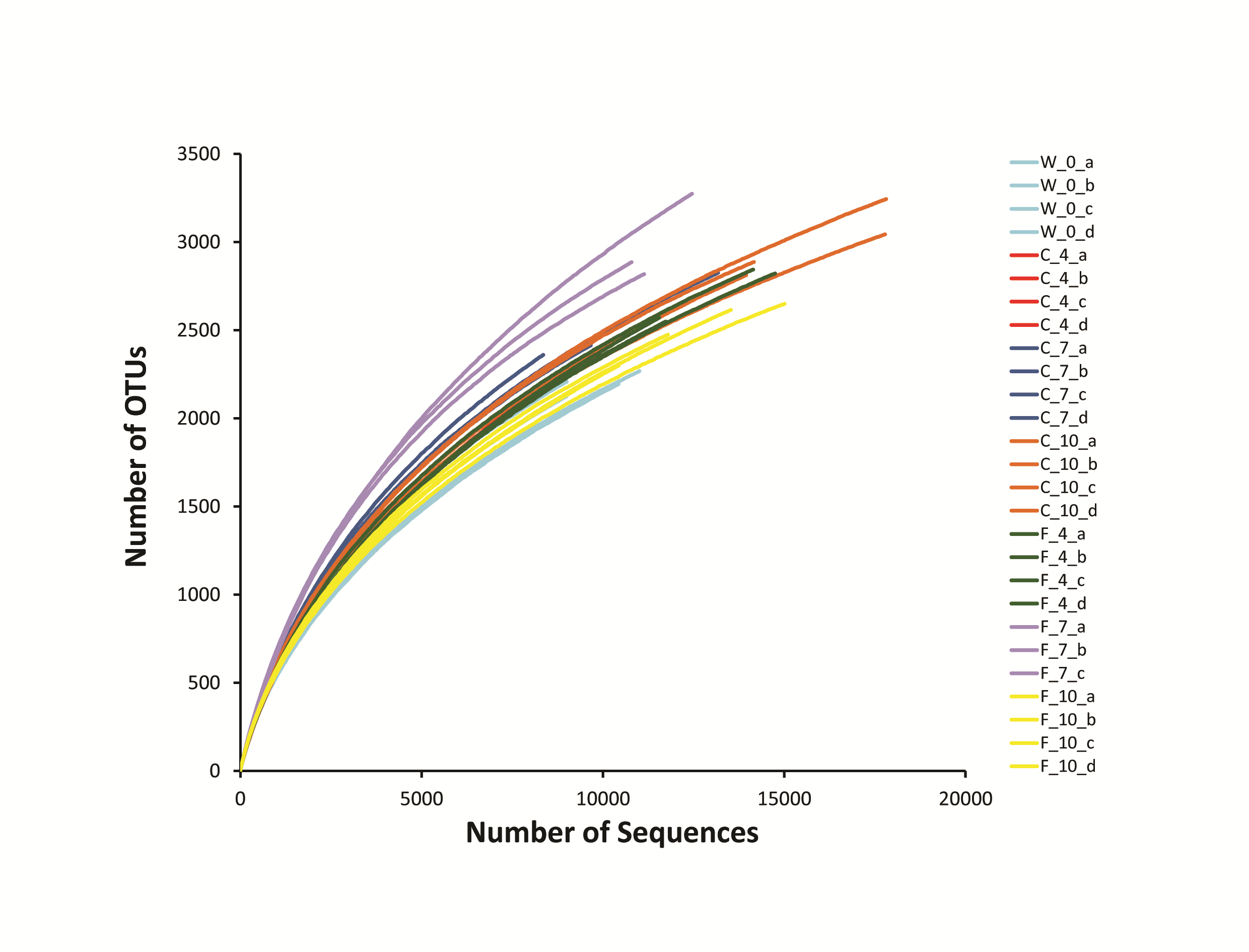


**Fig. S1.** **Rarefaction analysis of observed richness of the gut microbiome of *M. shangchengensis* individuals.** Curves were calculated at 3% dissimilarity level by MOTHUR program. The parallel samples from the same treatment groups are represented by the same color.


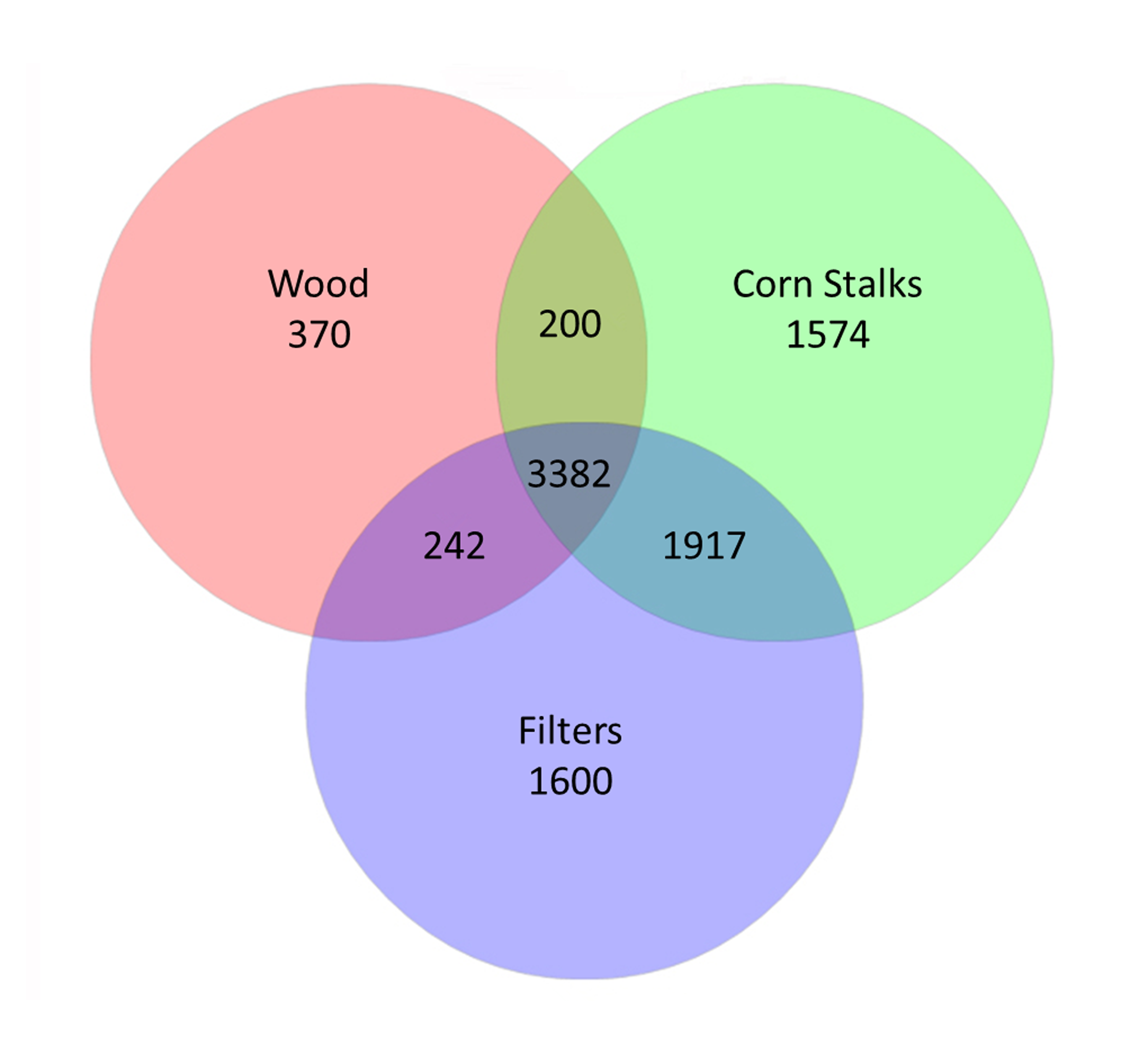


**Fig. S2. Venn diagram of different treatments observed overlap of OTUs at 3 % dissimilarity level.** The overlapping OTUs (3,382 OTUs) were shared by three different kinds of diets (wood, corn stalks and filters), which indicated that a core microbiome might exist in termite gut microbiota with diet variations.

**
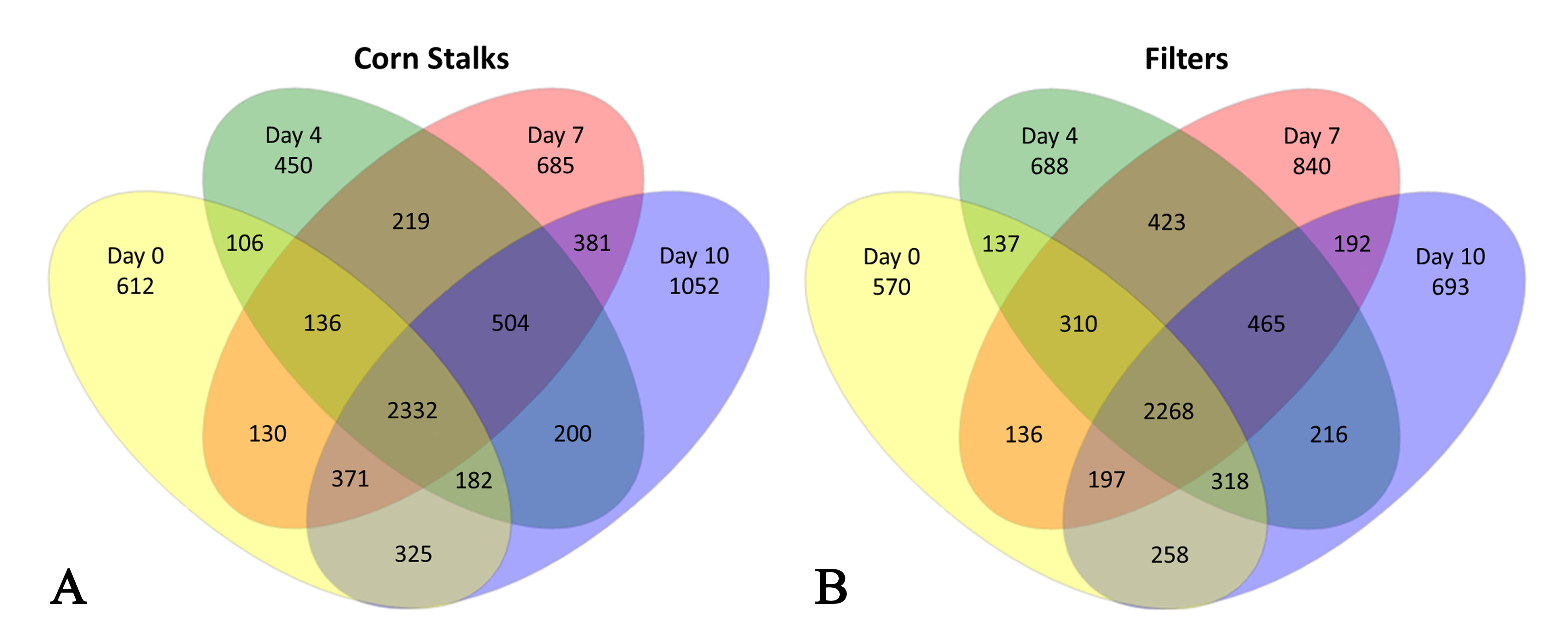
**

**Fig. S3. Venn diagrams of different feeding times observed overlap of OTUs at 3 % dissimilarity level for corn stalks (A) and filters dies (B).** Compared with the W_0 group (0 day), the overlapping OTUs were shared by different feeding times (4, 7 and 10 days) under corn stalks (2,332 OTUs) and filters (2,268 OTUs) substrates, respectively, which indicated that a core microbiome might existed in termite gut microbiota with temporal variations.


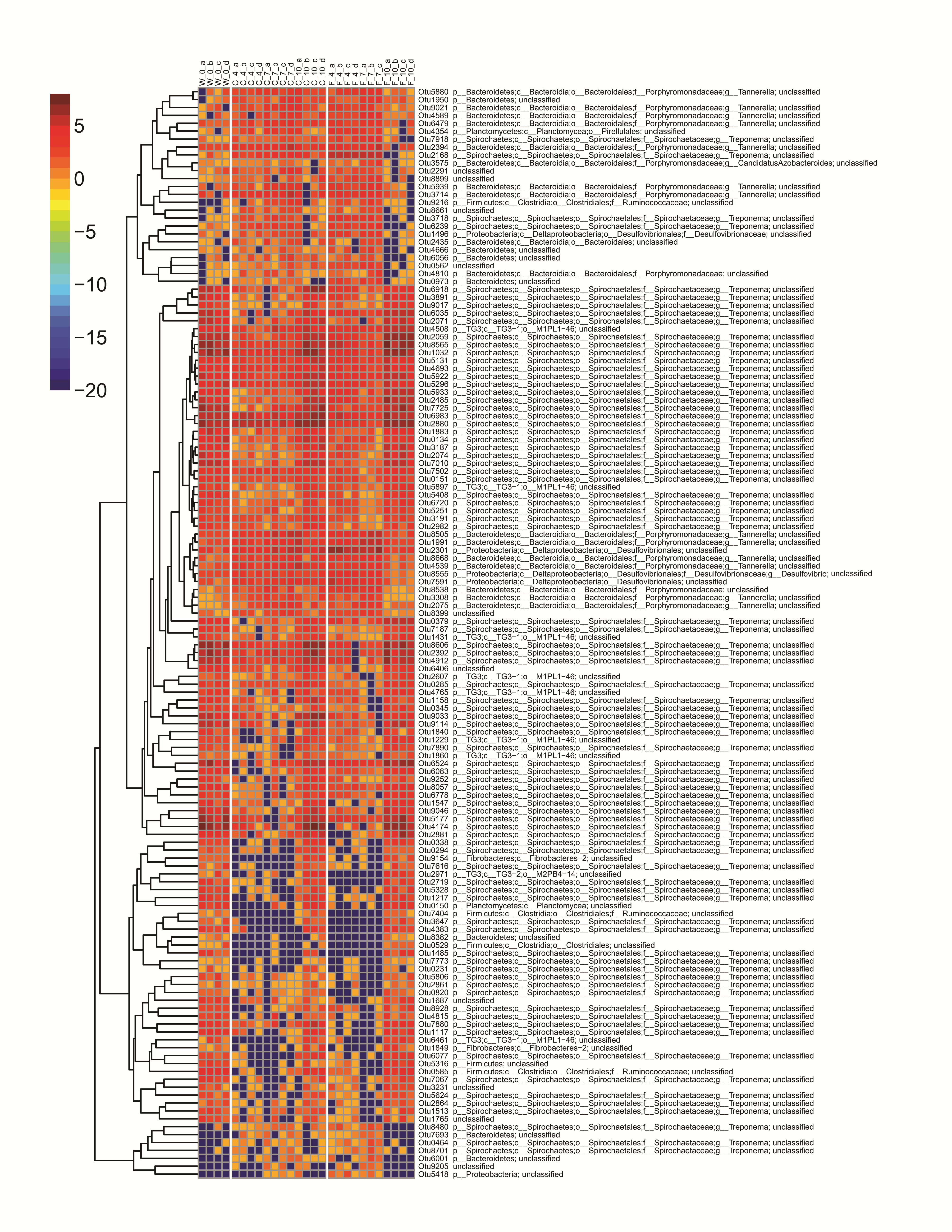


**Fig. S4. Heatmap of log2-transformed OTUs which had significant correlation with PC1 based on the unweighted UniFrac PCoA (Spearman |rho|>0.5, FDR q<0.2).**

**
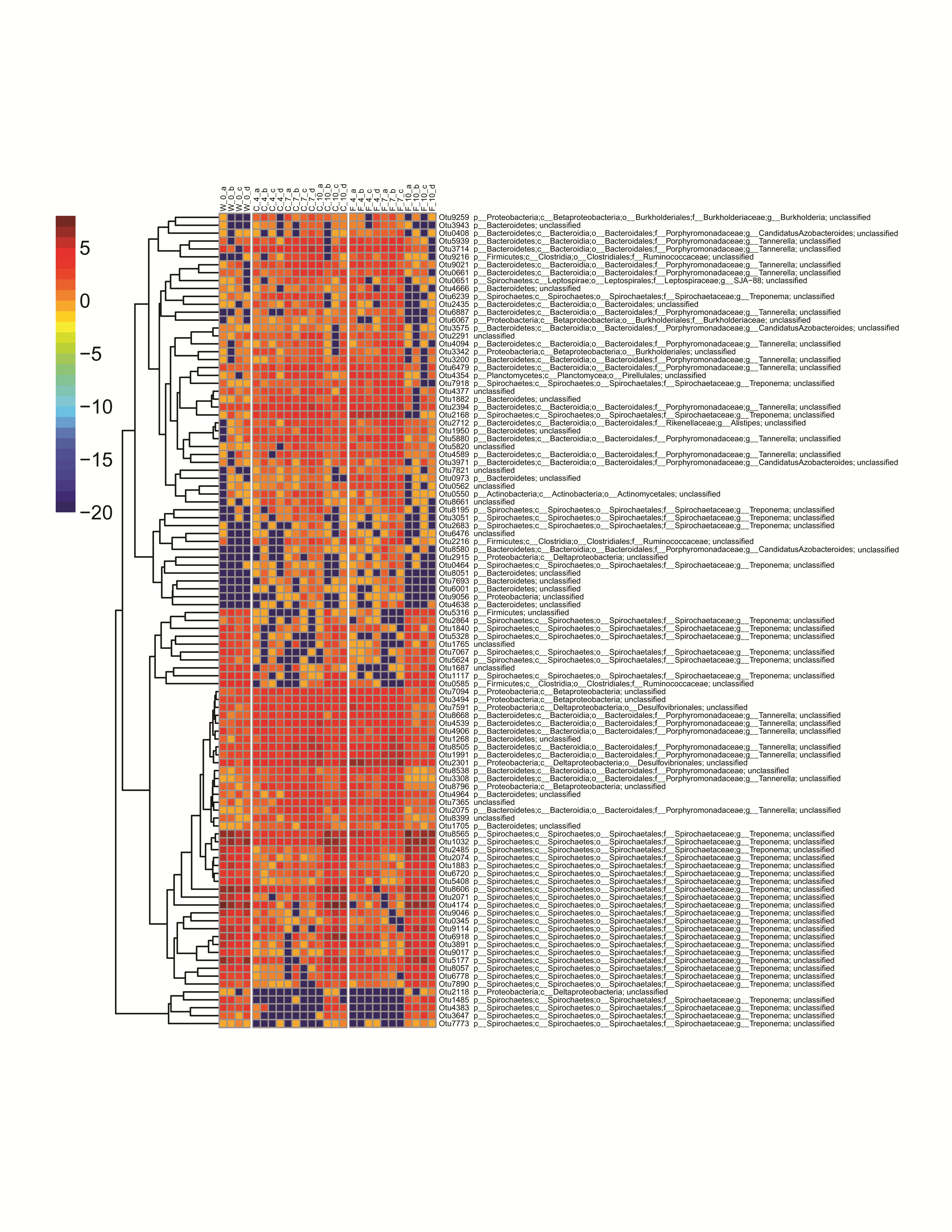
**

**Fig. S5. Heatmap of log2-transformed OTUs which had significant correlation with PC1 based on the weighted UniFrac PCoA (****Spearman |rho|>0.5, FDR q<0.2).**
